# Experiences of family caregivers of people with spinal cord injury at the neurosurgical units of the Komfo Anokye Teaching Hospital, Ghana

**DOI:** 10.1371/journal.pone.0284436

**Published:** 2023-04-21

**Authors:** Rumana Saeed Mohammed, Edward Appiah Boateng, Abigail Kusi Amponsah, Joana Kyei-Dompim, Timothy Tienbia Laari

**Affiliations:** 1 Department of Nursing, Faculty of Allied Health Sciences, College of Health Sciences, Kwame Nkrumah University of Science and Technology, Kumasi, Ghana; 2 Komfo Anokye Teaching Hospital, Kumasi, Ghana; 3 Presbyterian Primary Health Care (PPHC), Bolgatanga, Ghana; George Mason University, UNITED STATES

## Abstract

**Background:**

Spinal cord injury (SCI) often leaves affected persons with a lifelong demand for care. As a result, the effect of the condition goes beyond the injured person to affect family members and significant others who have to adjust their lives to provide care and support. However, little is known about the experiences of these family caregivers regarding the care of people with SCI in Ghana. Exploring their experiences would enhance the understanding of family caregiving of people with SCI and contribute to policy intervention.

**Methods:**

This qualitative descriptive phenomenology study used the purposive sampling method to select 10 family caregivers. Data were collected using a semi-structured interview guide through individual in-depth interviews. Written informed consent was obtained and interviews were audio-recorded and transcribed verbatim. Data were manually analysed following Colaizzi’s method of data analysis.

**Results:**

In all, 4 main themes emerged from the analysis of data (1) becoming a caregiver, (2) roles of the caregiver, (3) the burden of caregiving, and (4) coping strategies. The family caregivers provided vital assistance to their relatives with SCI and experienced physical and financial burdens as a result of the care. Due to the strains involved in the caregiving process, family caregivers adopted various strategies to cope with the situation.

**Conclusion:**

This study has provided evidence of the lived experiences of family caregivers of people with SCI in the Ghanaian context and further supports the findings of previous studies. Measures including training, counselling, and instituting social support services for family caregivers should be considered by the management of healthcare institutions to enhance the experiences of family caregivers.

## Introduction

Spinal cord injury (SCI) refers to damage to the spinal cord resulting from trauma such as a car crash or from non-traumatic disease or degeneration such as tuberculosis [1]. It is one of the worst medical conditions a person could experience, with its potentially lifelong effects going beyond the injured person to the family caregiver and significant others [2]. Globally, the prevalence of SCI is on the rise [3]. This is a result of road traffic accidents in developed countries and falls in developing countries [4].

The onset of SCI is devastating and affects the physiological, physical, social, economic, and psychological well-being of the individual. Injuries to the spinal cord, of either traumatic or non-traumatic aetiology, can lead to overwhelming and remarkable dysfunction and disability [5]. It is described as one of the greatest challenges to human beings mainly because SCI results in potentially severe disability and causes malfunctioning of major organ systems including the gastrointestinal, respiratory, urinary, autonomic nervous, and musculoskeletal systems [6]. The negative effects SCI has on multiple organ systems may result in several secondary complications and an increased mortality rate [6]. Adding to this, the inability of people with SCI to walk and the paralysis they experience, which sometimes affects their bladder or bowel control and sexual function, are some of the consequences of SCI [7]. Due to the neurological function of the spinal cord, any damage to the cord can result in total or partial sensory loss which can lead to life-long impairment or disability [8]. Neuropathic pain, autonomic dysfunctions and spasticity with muscle hyperactivity below the injury also may occur [8].

The World Health Organization (WHO) estimates that between 250,000 and 500,000 people live with SCI, with an annual global incidence of 40 to 80 cases per million population [1]. The majority (90%) can be attributed to road traffic accidents, violence, firearms, falls, and work hazards [4, 9, 10]. Traumatic SCI is progressively common following advancements in the means of transport and different high-risk occupations. Tuberculosis, malignant conditions, and Human Immunodeficiency Virus (HIV) have been identified as common causes of non-traumatic SCI/D in Sub-Saharan Africa (SSA) [1].

In the United States, with a population of 331 million people, an annual incidence rate of 54 cases per one million people or approximately 17,900 new cases of SCI are reported each year [3]. Males (78%) are more affected and the average age at injury is 43 years [3]. The recorded approximate incidence rate in South Africa in 2015 was 75.6 per million population [11] and 25.5 per million population in 28 low- and middle-income countries in 2013 [12]. It was found that in Nigeria, out of 468 cases of SCI recorded over 15 years, 312 (66.2%) were aged 40 years and below, with the peak incidence age recorded as 21–30 years [13], and the majority (70.1%) were males [13].

There has been progress in the management of the symptoms/side effects of SCI due to increasing knowledge of the mechanisms of the injury, its pathophysiology, and the role surgery plays. Yet, the proper management of symptoms/side effects of SCI requires financial resources that can place substantial demand on people with SCI, their families, and the community at large [14]. This is due to the high level of care required in the acute phase and the potential complications in the long term [15]. Although rehabilitation allows the individual to learn how to navigate through life with a SCI, the short length of stay at rehabilitation centers does not allow individuals with SCIs to fully adjust to the physical changes that occur post-injury. The implication is that the individual with SCI has less knowledge and expertise on how to take care of himself/herself to be less dependent on family members for assistance in activities which were formerly provided by healthcare workers during inpatient care.

The majority of individuals discharged from health facilities with SCI return to their habitations, mostly where they lived before the injury. Although some people with SCI can live alone successfully, others with more severe injuries usually require support with personal care to stay in the community [16, 17]. Individuals with SCI living in the community often are supported by unpaid informal caregivers, which primarily include family members [10, 15, 18]. Although the repercussions of SCI affect the entire family, usually one individual in the family assumes the primary caregiver role [19]. Existing studies have observed that the majority of family caregivers have close ties with their care recipients, mostly parents, children, siblings, or spouses of affected persons [20]. The majority of these family caregivers are females and the possible reason for this could be that females are considered traditionally to be caregivers. Neighbours and friends also have been found to offer some assistance to those with SCI living in the community [21].

Care rendered by family members is essential for the well-being of the person with SCI and to keep them within the community. Nonetheless, heightened physical and emotional changes have been reported among caregivers of people with SCI as a result of assuming the caregiving roles. People who provide assistance to individuals with SCI experience a lot of challenges and difficulties, especially in providing care for the long term [10]. It also has been reported that solving problems and performing roles associated with relationships and employment are among the difficulties informal caregivers experience [20]. Indeed, similar to persons with SCI, their caregivers also encounter problems with how to adapt themselves to the caregiving process [22]. Moreover, the early discharge of people with SCI often leaves the caregivers with limited time for learning caregiving skills, thus feeling unprepared to assume the caregiver role. Indeed, many caregivers do not receive sufficient support and this threatens the sustainability of the care provided [23]. To continue providing care, family caregivers require support so that their physical, emotional, and mental health needs are addressed.

Although healthcare workers are mainly responsible for caring for people with SCI while in an inpatient facility, family caregivers in Ghanaian healthcare facilities are actively involved in the care process during hospitalization. In the KATH for instance, family caregivers are actively involved in changing diapers, bathing, feeding, grooming, transporting, and turning their relatives with SCI. However, much is not known about the experiences of caregivers in Ghana. Therefore, this study explored the experiences of family caregivers of individuals with SCI in Ghana. Understanding their experiences is imperative as it would guide the development of interventions to support caregivers in Ghana.

## Materials and methods

### Study design and setting

The study was conducted using a descriptive phenomenology design to explore family caregivers’ lived experiences in caring for individuals with SCI. The design permitted the researchers to explore the experiences of the participants without interpreting them. The write-up of this paper was guided by the Consolidated Criteria for Reporting Qualitative Research (COREQ) checklist [24]. The study was conducted at the Komfo Anokye Teaching Hospital (KATH), an accredited hospital that provides tertiary health services mainly to serve the southern part of Ghana. However, the hospital also serves as a major referral facility for the Ashanti, Bono, Ahafo, Bono East, Northern, Upper West, Savannah, Northeast, and Upper East regions of Ghana. The hospital has 2 neurosurgical units made up of 2 wards: ward B1 and C1B with a total capacity of fifteen beds dedicated to the care of people with SCI.

### Study population, sampling technique, and sample size

The target population was comprised of family caregivers of people with SCI receiving health care at the neurosurgical units of the KATH. The inclusion criteria were family caregivers either present on admission or returning to the hospital after discharge for review, 18 years and older, and fluent in English or Twi language. Participants were recruited for the study through the purposive sampling technique [25]. The first author contacted the family caregivers face-to-face and informed them about the purpose of the study. In all, 10 family caregivers participated in the study. The sample size was determined by data saturation as no new data emerged from the responses after the 10^th^ participant. Also, no participant dropped out of the study.

### Data collection tool and procedure

A semi-structured interview guide S1 File designed by the authors in line with the objective of the study was used to collect data. The interview guide was pilot tested and divided into 2 sections (A and B). Section A of the guide was designed to collect participants’ socio-demographic data while section B collected data on the experiences of family caregivers of people with SCI. An audio recorder was used to record all the interviews while field notes were taken during interviews. Covid-19 protocols were observed by wearing face masks while maintaining physical distance throughout the interviews. The interviews were conducted between January and June 2020 by the first author, a female nurse with qualitative research experience, with no prior relationship with the participants. All the interviews were conducted at a convenient time for participants in the nurses’ room with no one else present. Interviews were started by asking participants to narrate their general experiences in caregiving for people with SCI before narrowing it down to specific questions based on the objectives of the study. Probing was used to elicit further details where necessary. Seven interviews were conducted in the Twi language and 3 in the English language. In all, 10 family caregivers were interviewed without repeat interviews and the interviews lasted 45–60 minutes.

### Data analysis and management

Colaizzi’s method was employed to manually analyse the data. The interviews conducted in the Twi language were translated into the English language by a Twi language expert, then audio interviews were transcribed verbatim by the first author. The first author read each transcript several times to get a general sense of the data and the statements about the experiences of caregivers were identified in each transcript. For each identified statement, meanings were formulated for them and then categorised into clusters of themes and sub-themes. An exhaustive description of the themes and sub-themes was written and condensed into short statements that represented the objective of the study. In all, 4 themes with subthemes were derived from the data. The transcripts were returned to the caregivers for their feedback and comments but none were recorded. The data were managed manually and soft copies of transcripts were saved on the personal computer of the first author. A unique participant number (P1…P10) was assigned to each transcript to conceal the identity of the participants and copies of the transcripts were also saved in a pen drive to prevent data losses.

### Trustworthiness

The framework developed by Lincoln and Guba was used to ensure trustworthiness in the research. Credibility was ensured through member checks where caregivers verified the data to ensure that the extracted statements represented their experiences. Transferability was ensured through thick description by describing the study setting, sample, and processes of inquiry. Confirmability was ensured as the researchers declared their personal biases, assumptions, and presuppositions and put them in a diary.

### Ethical considerations

Ethical approval for the study was obtained from the Committee on Human Research Publications and Ethics (CHRPE) of KNUST and the Institutional Review Board of KATH (KATH-IRB) with reference number RD/CR20/002. Written permission was also obtained from the management of the wards before the commencement of data collection. All participants of the study gave written consent by signing or thumb printing the informed consent form after receiving a briefing on the purpose of the study. Participation in the study was voluntary and participants could withdraw without consequences.

## Results

### Socio-demographic characteristics of participants

In all, 10 family caregivers participated in the study including 9 females and 1 male. Their ages ranged from 20–46 years (average = 34.2 years). Four were currently married while 6 were single. Regarding religion, 7 were Christians and 3 were Muslims. They have been involved in caregiving for 1–8 years (average = 4.1 years). Two were providing care for their mothers, 1 for a brother, 3 for their sisters, 1 for a son and 3 for their spouses. The socio-demographic characteristics of the participants are presented in S1 Table.

### Main findings

In all, 4 main themes emerged from the data including (1) becoming a caregiver, (2) roles of the caregiver, (3) burden of caregiving, and (4) coping strategies. These themes with their subthemes are presented in Table 1 below.

**Table 1:** Themes and sub-themes generated from the data.

Major themes	Subthemes
Becoming a caregiver	General concerns Social changes
Roles of the caregiver	Activities for daily living Instrumental activities for daily living
The burden of caregiving	Physical burden Financial burden
Coping strategies	Source of strength Hope and religious practices Social support

#### Becoming a caregiver

This theme has 2 subthemes; general concerns, and social changes. The theme describes how caregivers became aware of their new roles and how the roles brought them closer to their family members and developed compassion for them and how people with SCI appreciated their efforts.

*General concerns*. This subtheme describes aspects of caregiving and hospitalisation that participants identify as problematic. A notable concern in this subtheme was the reaction to the sudden occurrence of injury, patient symptoms, unfamiliar environment, and physical demands of in-patient care. The occurrence of SCI was noted to be sudden, unplanned and unexpected. Most participants noted that a person with SCI was involved in a road traffic accident or a fall which resulted in the injury:

*“He was on the top of a moving articulated truck at night and I think it hit something so he fell from the truck onto the ground and that was how he got here with the injury”*

*P2*.

Participants received the news of the injury with shock and disbelief as they never expected this injury to have happened.

*“It was too sudden and I never dreamt that something like this would happen to him*. *I did not believe it at first until I saw him at the hospital and realised that it was true after all*. *I just stood there not knowing what to do”*

*P1*.

In addition, participants were anxious about the consequences of the injury as it was their first time experiencing such an event.

*“I had never seen something like that before and I did not know what to do*. *Looking at how active he was*, *I was really worried if he would remain the same after discharge”*

*P8*.

The sudden occurrence of SCI resulted in the sudden entry into the caregiving role which participants were not ready for but had no other option than to take up. In addition, it was the first time many participants had to care for a relative within the hospital setting.

*“I was just not ready for this but it happened and there was no one to stay with him so I had to do all that in addition to my work*. *It has been a very difficult time for me because I have never done this”*

*P9*.

Participants were also concerned about the symptoms a person with SCI experienced. In this regard, the agitated and restless states of the person with SCI were sources of worry for participants:

*“His hand became [so] stiff that he could not move it*. *It was the first time I saw something like that and anytime I spoke with the nurses*, *they said he will be okay”*

*P6*.

Also, a participant was concerned about incontinence as it required changing diapers frequently:

*“I had to change his diapers frequently because he could not do it by himself*. *Even when the toilet is coming*, *he cannot feel it”*

*P4*.

*Social changes*. Participants indicated the changes they experienced in their social lives due to their role in providing care to people with SCI. They mentioned the fact that the caregiving role has taken all their time and they cannot find time to socialize with friends and loved ones. These were expressed in responses like:

*“I can’t go anywhere anymore because I always have to be in the hospital… I spend all the time visiting the hospital for management of my husband and cannot find time for fun”*

*P4*.

*“I enjoyed travels and holidays with my family but now I have to limit myself to take care of my father in the hospital”*

*P9*.

However, some participants also expressed positive things that they have experienced due to the changes brought to them by their caregiving role.

*“I have come into contact with many people who have blessed my life during this period of caregiving*. *I think my husband’s injury has brought the family and friends together*. *Even though his condition has limited my life*, *I believe it has also brought a lot of people into our lives*”

*P10*.

#### Roles of the caregiver

This theme describes the various roles caregivers played in caring for people with SCI. As people with SCI were largely dependent on their caregivers to perform certain activities, the caregivers were readily available to assist with the roles of caregiving. The 2 subthemes that emerged from this theme were activities for daily living and instrumental activities for daily living.

*Activities for daily living*. The caregivers were greatly involved in assisting people with SCI to perform their activities of daily living. Participants described how they assisted people with SCI to perform activities such as bathing, dressing, feeding, and bladder and bowel management.

“*Because of his condition*, *he is not able to do anything*. *I bathe and feed him; I mean everything that he used to do I am doing them for him now”*

*P4*.

*“I do everything for her because she cannot help herself; from feeding to bathing and helping her to pass urine or toilet is my responsibility”*

*P6*.

*“It has not been an easy task for me at all*. *I clean him*, *bathe and dress him all the time*. *He cannot lift himself to even urinate or visit the toilet*. *I have to always do things he needs to live for him”*

*P10*.

*Instrumental activities for daily living*. Participants were found to be performing instrumental activities for daily living in their roles as caregivers. These roles involved transporting the person with SCI, cooking, managing the finances of people with SCI, and housekeeping. These were expressed in statements like:

“*Since he was discharged from the hospital*, *I always carry him from home for review all the time*. *Apart from that*, *I do the cooking and run errands for him”*

*P5*.

*“I am doing many things for him that I cannot even count*. *Apart from other personal things*, *I am now taking care of the home and sending him to wherever he wants to go*. *I am also the one who manages his business and money”*

*P8*.

*“Apart from feeding and dressing him*, *I cook for her and carry her from one place to the other anytime there is need”*

*P7*.

#### The burden of caregiving

This theme describes the challenges the caregivers experienced while caring for people with SCI. As caregivers were constantly supportive of the care of people with SCI, they expressed the challenges they experienced as a consequence of constantly caring for people with SCI. This theme has the physical burden and financial burden as subthemes.

*Physical burden*. The physical burden reported by participants in the study included body pain, sleeplessness, tiredness, and sickness due to the caregiving role. Many complained that the caregiving role demanded a lot of energy as they have to turn and lift the individual with SCI from time to time. This drained them of their physical strength, made them tired, and sometimes, caused them to be sick.

*“I always get tired because I have to turn him all the time and*, *at night*, *I cannot sleep well because he always needs one help or the other”*

*P5*.

*“I am physically weak these days because of the work involved in taking care of someone with SCI*. *I have to do almost everything for him and sometimes I fall sick due to this work”*

*P9*.

*Financial burden*. Admission to the hospital came with a significant financial burden for caregivers of individuals with SCI. This was acknowledged by participants who pointed out that this burden results from their inability to carry out their normal business and make money as before. At the same time, the care of the person with SCI demanded more financial commitment.

*“Now I cannot work as before because I spend all my time taking care of him*. *Because of this*, *my financial situation has become worse*. *Meanwhile*, *I need more money to take care of him*. *For now*, *I am really*, *really burdened financially”*

*P1*.

Meeting the financial demands of in-patient stay also required caregivers to make hard decisions which were problematic for them. Some decisions were related to deciding whether to use the money available to buy food or medications.

*“Sometimes the money available is just not enough so I have to choose whether to use it to buy medications or food*. *But you see*, *the two are important but I usually get confused about what to do”*

*P7*.

#### Coping strategies

Coping strategies as a theme has sources of strength, hope and religious practices, and social support as subthemes. Participants shared various ways they use to cope with the caregiving situation. Some indicated that in addition to other coping strategies, they cope with the pain and the difficulty in caregiving by finding a little time within their schedule for their personal needs. This, they said, was after they realized that their situation would not end soon and hence, accepted it as part of their normal life, that is, to live to care for the person with SCI.

*“The situation has become a normal part of my life and I have to live with it*. *No matter what*, *life must go on so you have to also get time for your personal care”*

*P10*.

*“Now I have come to know that this is our situation and we must live with it*. *My husband and I have promised each other to live in bad or good times and here is a bad time for us to show commitment to each other*. *So*, *we accept whatever we are going through and I cannot give up on this situation*. *I am taking care of my husband but I still live my normal life so both of us are not worried at all”*

*P8*.

*Sources of strength*. Participants drew strength from various sources to keep them moving despite the frustrations associated with caregiving. While most of them indicated their source of strength was God, others draw their strength from friends and loved ones.

*“I believe that God will answer our prayers one day and he will get healed*. *I am confident in God and when I pray*, *I feel strong to do anything”*

*P6*.

*“My friends have not left me in this fight*. *They have supported me with both money and words*. *Their constant visits keep me strong and hopeful”*

*P7*.

A few participants revealed that they depended on alcohol to cope with the situation of caregiving. They asserted that after hoping for a long time to see the recovery of their relatives with SCI which never came, they resorted to alcohol as a way to forget their problems.

*“At a point*, *I became worried and fed up with myself*. *It is alcohol that I depend on to forget my sorrows”*

*P1*.

*Hope and religious practices*. From the onset of the injury, participants hoped that the injured persons would get well soon and be discharged as they perceived that the SCI was acute and limited to the physical wounds that they saw. This hope appeared to be the force that drove participants to continue purchasing items needed for the care of people with SCI. Thus, they seemed prepared to go the extra mile to achieve this recovery.

*“As soon as I am called to buy any medications*, *I just leave immediately if only I have the money available*. *I just hope that he would get better so that we go home as soon as possible”*

*P2*.

Participants noted that strength from God allowed them to undergo the caregiving journey. Participants indicated that it would have been difficult if they had not relied on God for strength and guidance through the hardships and reality they experienced. Therefore, throughout the caregiving journey, they maintained their faith in God but wished that the recovery would be faster for them.

*“ I just see God as my source of strength through this period because all that happened was so shocking and frightening*: *losing a neighbour and still coming to take care of him in the hospital really requires supernatural strength”*

*P3*.

The belief and faith expressed by participants are also associated with the performance of various religious practices. These religious practices included praying and fasting with the hope of an expedited recovery. It was noted that anytime they visited the people with SCI, they pray with them. Participants also shared that they sang Christian songs to comfort themselves and the people with SCI.

*“As early as 2 am*, *I would wake up and pray continuously and sometimes fast all with the hope that my husband would get better*. *Even after I leave his room*, *I continue praying and would sing some songs by his side because he really likes music”*

*P5*.

*Social support*. Participants obtained support from their families to meet the needs of people with SCI. This included financial support and frequent calls to follow up on the state of the person with SCI as well as the caregiver.

*“The entire family helps to take care of some of the financial issues and they always call to check up on how I am faring*, *and the old man too”*

*P2*.

*“My family has been supportive in some ways like raising funds for the medical bills and they always calling to find out what is happening”*

*P10*.

Another form of family support was taking charge of participants’ responsibilities in their absence at home. Thus, some family members are involved in taking care of participants’ children as well as operating their businesses in their absence.

*“In my absence*, *other family members take care of my child*. *In the mornings when I leave home*, *they bathe the child and take him to school and it helps me a lot*

*P3*.

*“I did not want anyone to open the shop but because we needed the money*, *I had to allow some of the family members to open the shop in their free time so they could sell the items there”*

*P6*.

Family members also supported participants with religious practices such as prayers and fasting:

*“We all pray and fast*, *I mean the family do that too because God is everywhere and could hear us*. *I do mine here and they do theirs too at home*. *Some of them go to prayer camps for prayers too because we want him to survive and live so that we could return him to his relatives”*

*P8*.

*“I wish this had not even occurred at all but it did happen but there is nothing that prayer cannot solve*. *My sister prays with me most of the time*. *She even fasts”*

*P4*.

In addition, participants received various forms of encouraging words from family members.

*“Sometimes when I speak with the people at home and they tell me everything will be fine*, *I feel like they can see what is happening here and I was okay*. *If not*, *I wondered how I could have gone through this hard time”*

*P9*.

## Discussion

Family caregivers of individuals with SCI were found to be concerned with the sudden nature of their positions as caregivers. It was noted that the majority of the participants did not expect the condition in which their family members were found and as a result were concerned about the sudden occurrence of the injury, symptoms, and not being familiar with the hospital environment. Another concern found to be associated with becoming a caregiver of individuals with SCI was the social changes in their lives due to their family caregiving roles. These concerns were related to how the caregivers had to adjust their normal lives to take up the role of caring for their relatives with SCI. The thinking of difficult circumstances surrounding caregivers of individuals with SCI can negatively affect their health, thereby preventing them from effective caregiving activities. These findings are therefore significant as they call for the need to pre-counsel family caregivers to avoid them experiencing shock out of depression.

In this study, caregivers of individuals with SCI played multiple roles as caregivers. On a daily basis, they assisted people with SCI in carrying out activities of daily living. They mostly played a role in bathing, brushing teeth, dressing up, administering medications, toileting, and transferring people with SCI. These roles were expected of the family caregivers considering the condition of people with SCI which makes it difficult for them to exercise control over their bodies. Although these roles could be played by nurses, family caregivers performed them due to the limited number of nurses compared to the workload in the ward. This finding is in support of the findings of a previous study [26] which reported that the basic role of family caregivers was assisting people with SCI with activities for daily living. Our study also reports that caregivers of individuals with SCI were involved in instrumental activities for daily living such as transportation, shopping, preparing meals, and managing the finances of the care recipient. This finding is consistent with the finding of earlier studies [27, 28].

The findings of our study revealed that family caregivers of persons with SCI experience physical and financial burdens. The physical burden experienced by caregivers in this study was related to the increasing roles that family caregivers had to assume as they provide care for people with SCI. Participants revealed that they experienced body pains and tiredness in the course of attending to the needs of the care recipients. Our finding resonates with the findings of previous studies [29–31] that have suggested that when people take up caregiving roles, they find themselves in an unexpected career that they have to perform for sick or disabled persons. The sudden occurrence of the injury implied unexpected and sudden entry into the caregiving role. This was observed to be associated with a lack of options and unpreparedness. These unexpected and demanding roles of caregiving put some level of strain on these family caregivers as this study found that participants experienced financial difficulties as they could not get time to work and earn income. These together brought a financial burden on caregivers who had to borrow money to survive. The stress brought to caregivers as a result of financial burden was considered the most worrying and the hardest that they could bear in caring for individuals with SCI. This finding of our study is consistent with that of Rodakowski et al. and Boschen, Tonack, & Gargaro [16, 32] that concluded that family caregivers experience financial constraints.

The study found that family members who provided care to their relatives with SCI adopted some strategies to cope with their challenges. Through their religious practices, hope, and the support they received from relatives, friends and loved ones, caregivers coped with the challenges. Caregivers in this study relied on religious practices and hope. This, for caregivers, was known to connect them to God who is the maker of all things. This finding was expected considering the religious nature of Ghanaians and the participants of the study in particular. The finding corroborates the finding of Charlifue et al. and Chan, Lee, & Lieh-Mak [30, 33] who found that family caregivers of people with SCI coped by having faith in God. Religious people have faith in God as the power behind everything either bad or good. This was the position of participants in the study as they lived with their hope in God that the people with SCI will one day be fully recovered and assume normal life activities. This was found to keep caregivers in their role despite the physical and financial burden they encountered.

Our study also uncovered that family caregivers coped with their situation by relying on the support they got from relatives, friends and loved ones (social support). In line with the finding of our study, social support such as peer support groups is a key source of help for caregivers. This finding of our study further supports that of a previous study [20] which found the need for support to prepare relatives of people with SCI for their caregiving. This support would help in the emotional and physical health of caregivers.

### Limitations of the study

The authors acknowledge some limitations in the study. Only one male caregiver and three Muslims were included in the study. The inclusion of more male and Muslim caregivers could generate new findings that could alter the findings of the study. This notwithstanding, the study provides useful information on the experiences of caregivers of people with SCI in Ghana.

## Conclusion

The study has revealed that family caregivers of individuals with SCI transitioned into their positions without adequate preparation. They however performed roles relating to activities for daily living and instrumental activities for daily living. Due to the roles of caregiving, family caregivers experience both physical and financial burdens as they get more involved in caregiving roles at the expense of any economic activities. Support from family members and friends as well as hope and religious practices form the main coping strategies of family caregivers of individuals with SCI. The findings call for comprehensive training for family caregivers before they assume the duties of caregiving. Social support systems for family caregivers of individuals with SCI are also necessary to reduce the financial burden associated with caregiving.

## Supporting information

S1 File Semi-structured interview guide. (DOCX)

S1 Table Socio-demographic characteristics of participants. (DOCX)

## Abbreviations

IADL: Instrumental Activities for Daily Living
KATH: Komfo Anokye Teaching Hospital
MOH: Ministry of Health
SCI/D: Spinal Cord Injury/Disease
WHO: World Health Organization
