# Supplementary material for: Experiences of family caregivers of people with spinal cord injury at the neurosurgical units of the Komfo Anokye Teaching Hospital, Ghana
Source: PLoS One. 2023 Apr 21;18(4):e0284436. doi: 10.1371/journal.pone.0284436 (PMC10121035; doi:10.1371/journal.pone.0284436)
Supplement: S1 File — (DOCX) [file pone.0284436.s001.docx]

**Semi-structured interview guide**

**Section A: Socio-demographic data**

Participant number___________________________

Marital status ______________________________

Religion__________________________________________________________________

Duration of care____________________________________________________________

**Section B: Experiences of family caregivers of people with spinal cord injury**

1. Kindly tell me your experience of becoming a caregiver of someone with spinal cord injury

**Probes:**

- What are your general concerns as a caregiver?
- How has the caregiving change your life?

1. Please tell me what you do as a caregiver of someone with spinal cord injury

**Probes:** Tell me;

- The activities do you perform on daily basis for the patient
- Any other activities you do for the patient

1. Tell me how caring for someone with spinal cord injury affected your life.

**Probes:**

- Your physical health
- Your finances

1. How do you cope with your experience as a caregiver of a person with spinal cord injury?

**Probes:**

- How do you cope with the physical burden of caregiving?
- How do you cope with the financial burden of caregiving?
