# Supplementary material for: Experiences of family caregivers of people with spinal cord injury at the neurosurgical units of the Komfo Anokye Teaching Hospital, Ghana
Source: PLoS One. 2023 Apr 21;18(4):e0284436. doi: 10.1371/journal.pone.0284436 (PMC10121035; doi:10.1371/journal.pone.0284436)
Supplement: S1 Table — (DOCX) [file pone.0284436.s002.docx]

**Table 1. Socio-demographic characteristics of participants**

| Participant number | Marital status | Religion | Duration of care (years) |
| --- | --- | --- | --- |
| P1 | Single | Christian | 1 |
| P2 | Single | Christian | 5 |
| P3 | Married | Christian | 8 |
| P4 | Married | Muslim | 3 |
| P5 | Single | Christian | 1 |
| P6 | Single | Christian | 2 |
| P7 | Single | Christian | 4 |
| P8 | Married | Muslim | 8 |
| P9 | Married | Muslim | 4 |
| P10 | Single | Christian | 5 |
